# Emerging risk identification in the food chain using text mining–supported monitoring of food safety news: insights from 2022 to 2023

**DOI:** 10.1038/s41598-026-50890-8

**Published:** 2026-04-27

**Authors:** Zsuzsa Farkas, Andrea Zentai, Máté Farkas, Krisztián Vribék, Szilveszter Csorba, Erika Országh, Orsolya Strang, Tekla Engelhardt, Miklós Süth, Ákos Józwiak

**Affiliations:** 1 Institute of Food Chain Science, Department of Digital Food Science, University of Veterinary Medicine Budapest, István str. 2., Budapest, 1078 Hungary; 2 National Laboratory of Infectious Animal Diseases, Antimicrobial Resistance, Veterinary Public Health and Food Chain Safety, University of Veterinary Medicine Budapest, Budapest, 1078 Hungary; 3 Institute of Food Chain Science, University of Veterinary Medicine Budapest, István str. 2., Budapest, 1078 Hungary

**Keywords:** Emerging risk identification, Food chain risks, Data analysis, Topic detection, Text mining, Environmental sciences, Risk factors

## Abstract

The identification of emerging risks in the food chain is essential for proactive food safety management in a rapidly evolving global environment. In this study, we applied a structured emerging risk identification framework supported by text mining of food safety news collected from the Europe Media Monitor (EMM) to analyse signals from 2022 to 2023. The workflow combines automated news collection, topic detection, and expert evaluation within a multi-stage filtering system. Using this approach, 164 emerging risks and drivers were identified during the study period. Chemical risks—including pesticide-related issues, per- and polyfluoroalkyl substances, and microplastics—were among the most frequently detected topics, while sustainability-related developments such as alternative proteins and novel packaging technologies emerged as major drivers of change. Comparison with the 2020–2021 period indicates a marked increase in signals related to chemical contaminants and sustainability. These findings highlight the value of combining automated text mining of food safety news with expert-based evaluation to support timely emerging risk identification in the food chain.

## Introduction

Emerging risk identification (ERI) in the food chain remains a highly relevant and essential field in our rapidly changing world. The food chain is constantly evolving due to factors like climate change, globalization, changing consumer behavior, and emerging pathogens. These can introduce new or intensified risks, making early identification essential for protecting public and environmental health.

In such an incredibly dynamic environment, governments must be prepared for expected and unexpected changes in global food systems and the potential impact certain changes could have on food and feed safety. Anticipation and preparedness for potential disruptions are key to building more resilient agrifood systems^1^.

In this context, particular attention is paid to so-called *signals*, understood as early indications or pieces of information that may suggest the emergence of new risks or changes within the food system. These signals represent preliminary outputs of the monitoring process and do not yet constitute emerging issues or risks. Instead, they provide the basis for subsequent expert evaluation, which may lead to their classification as emerging issues, potential emerging risks, or emerging risks, or to their interpretation as indicators of underlying drivers.

Recognizing the need for ongoing attention to effectively anticipate and respond to new and shifting threats within the food chain, along with the challenges due to the unpredictability of emerging food safety risks, food safety institutions worldwide make efforts to develop procedures assisting them in timely identification of such risks. In a recent report by the Food and Agricultural Organization of the United Nations (FAO), Siligato et al. provide an overview of food safety foresight approaches developed by governments, international organizations, research institutes, and the private sector^1^. Within this context, the framework applied in the present study has been presented as a case example in the FAO report, highlighting the system developed at the University of Veterinary Medicine Budapest as an operational and applied approach to emerging risk identification.

Furthermore, the system contributes to European-level activities through the European Food Safety Authority’s (EFSA) Emerging Risk Exchange Network (EREN), where identified European Union (EU) relevant potential emerging risks are regularly shared and discussed among network members and stakeholder organizations. This process supports subsequent risk assessment or risk management considerations, illustrating the practical relevance of the monitoring approach within coordinated food safety governance, including cases where issues such as food fraud may have implications for food safety. Similar efforts to develop structured frameworks for emerging risk identification have also been supported at the European level, for example through the EFSA-funded DEMETER project^2^, where a conceptual framework for an Emerging Risks Knowledge Exchange Platform (ERKEP) was developed.

ERI systems have been developed at both international and national levels, often combining horizon scanning, data analysis, and expert evaluation. At the European level, structured frameworks enable cross-country information exchange and coordinated identification of emerging issues. EFSA, for instance, has set up a complex system already in 2010 involving extensive data collection and international cooperation through EREN^3^. In parallel, several national-level systems have been described, integrating diverse data sources and expert knowledge to identify context-specific signals/emerging risks, including structured national strategies (e.g. the Spanish Agency for Food Safety and Nutrition—AESAN^4^), data-driven analytical workflows, and institutionalized horizon-scanning systems (e.g.the Emerging Risk Identification System (ERIS) platform of the Institute of Environmental Science and Research (ESR) in New Zealand^5^, the Vigilance and Intelligence Before food issues Emerge (VIBE) system in Food Standards Australia New Zealand (FSANZ)^6^. However, relatively few ERI systems are described as continuously operating, systematically maintained, and consistently applied over extended periods. In this context, the added value of the present study lies not primarily in methodological novelty, but in demonstrating a sustained, operational ERI workflow that contributes to and complements broader European-level efforts. National systems such as the one presented here play a critical role in detecting locally relevant emerging risks in a timely manner and feeding them into international knowledge exchange mechanisms, thereby strengthening collective early warning capacity.

Text mining (text data mining, text analytics) can be a beneficial tool to aid emerging risk identification, with the processing of large amounts of textual information. The main objective of text mining is to extract valuable information, uncover patterns, and generate knowledge from large volumes of text. Utilizing natural language processing (NLP), machine learning, and statistical techniques, text mining models analyse textual data through multiple stages. These models play a key role in transforming unstructured text into structured insights, enabling organizations to make informed, data-driven decisions. By leveraging advanced NLP and machine learning techniques, text mining reveals hidden patterns within textual data, offering valuable information and fostering innovation across various industries.

Several food-related studies demonstrated the utility of such methods in different research areas across the food chain^7–11^. Carneiro et al. applied natural language processing and machine learning to large collections of food security reports to identify key global drivers of food insecurity, demonstrating how text-based data sources can be transformed into structured knowledge for decision-making and strategic planning^12^. Similarly, Chen et al. reviewed visual analytics methods developed for food safety risk analysis, highlighting the growing role of big data techniques and interactive data visualization in detecting patterns and supporting risk assessment within the food system^13^. Furthermore, predictive modelling approaches have been applied to food security monitoring: Foini et al. developed machine-learning models combining food consumption data with information on conflicts, weather events and economic shocks to anticipate short-term trends in food insecurity, illustrating the potential of integrated data sources for anticipatory analysis and early warning systems^14^. Together, these studies demonstrate the increasing relevance of advanced data analytics for extracting actionable insights from large and heterogeneous datasets related to food systems^15^.

While these recent developments have shown promise for the identification of emerging risks as well, they focus on different specific areas of the food sector. In contrast, the present work focuses on supporting the early identification of signals and trends relevant to food safety through continuous monitoring. The integration of text mining into the process of emerging risk identification provides opportunities for enhanced and more efficient emerging risk identification. The relevance of this work increases as current systems primarily rely on expert knowledge and scientific literature, with a continuous shift towards exploitation of advanced data analytical methodologies^3^.

In our previous study, we introduced a systematic process management system designed to identify and manage emerging risks in the food chain^16^. This system aimed to address the complexities and uncertainties inherent in emerging risk identification. It provided a structured process, applicable data analysis methods, and a practical analysis of food chain risks from 2020–2021, covering areas from microbial safety to climate change.

In our present study, building on the experience gained since the previous publication, we analyse the emerging risks identified in 2022 and 2023 using the previously developed process management system. The study demonstrates the application of text mining–supported screening of food safety news combined with expert evaluation within a structured workflow for emerging risk identification in the food chain. By applying this framework to recent data, we provide an updated overview of emerging risks and drivers affecting the food system, and evaluate the practical operation of the monitoring system in a rapidly evolving information environment. Compared to this previous work, the present study reflects a more mature and extended application of the system. While the earlier study focused on establishing the methodological framework and demonstrating feasibility, the current work applies the system to a new dataset (2022–2023), enabling the analysis of temporal patterns and evolving risk dynamics, and incorporates methodological refinements based on accumulated operational experience.

The aim of this study is to present the emerging risks identified in 2022 and 2023 using our process management system and our traditional topic detection methods, and to discuss the results, experiences, and limitations of these methodologies in the context of the food chain, with a focus on the enhancement of the emerging risk identification process. By integrating process management approaches with advanced data analytical methods, we aim to provide a more robust and scalable framework for emerging risk identification, ultimately contributing to improved food safety and public health outcomes.

## Materials and methods

### Overview of the system employed for emerging risk identification

**Fig. 1:**
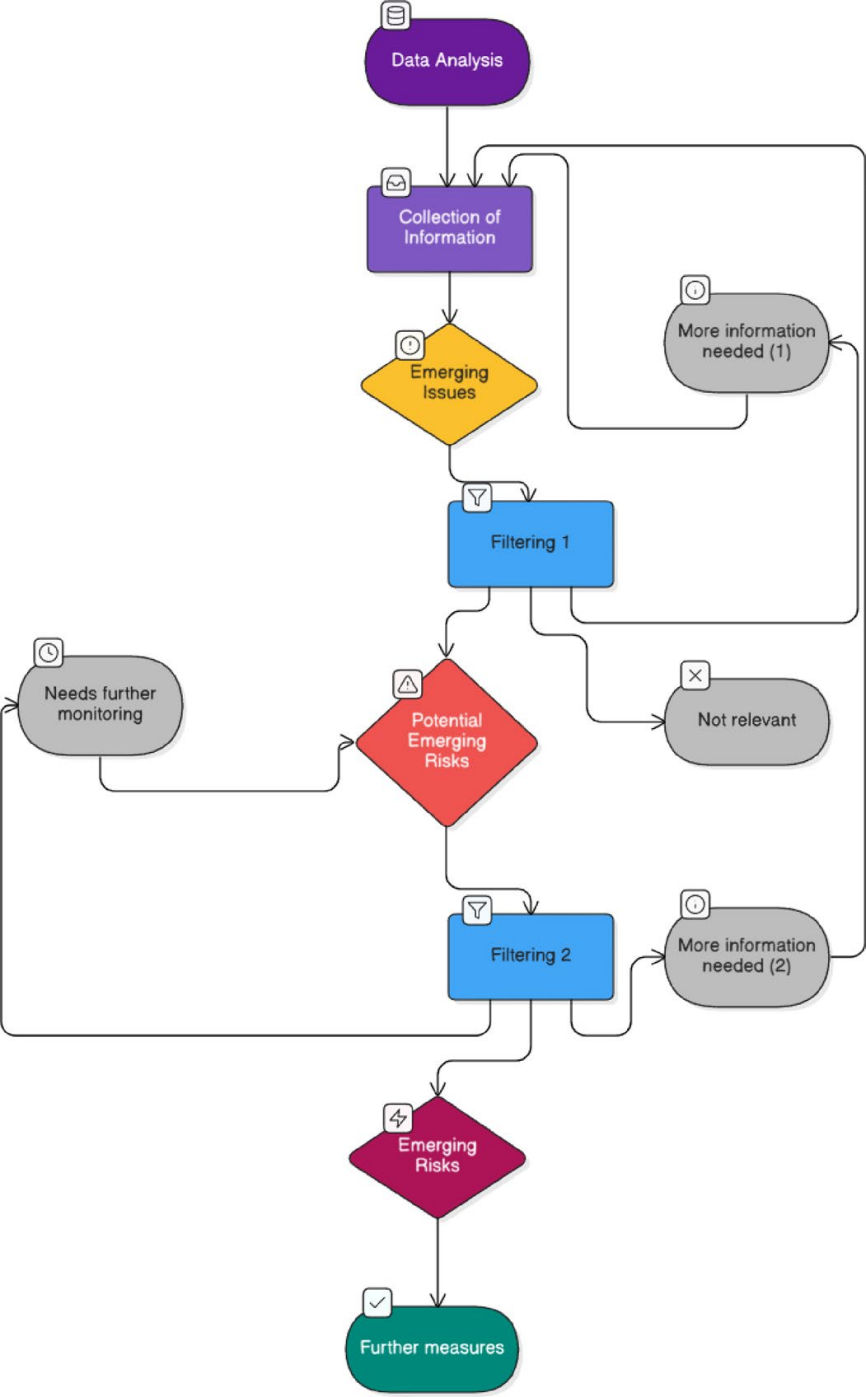
Schematic illustration of the applied workflow for emerging risk identification.

The process management system employed for the identification of emerging risks in the food chain for the years 2022 and 2023 is consistent with the system detailed in our previous study^16^. In essence, the identification process involves the collection and structured filtering of relevant data and information sources (Fig. 1). This process consists of three phases:

- PHASE I: Selection of emerging issues from the gathered data and information.
- PHASE II: Identification of potential emerging risks from the selected emerging issues.
- PHASE III: Selection of emerging risks that require further measures from the identified potential risks.

The framework, shown in Fig. 1, is a versatile workflow used successfully by our team. As risks evolve with new data and insights, the process uses different terms to categorize the stages of identification. An “emerging issue” is a newly identified concern posing potential food or feed safety risks, warranting further investigation due to initially limited information. An emerging risk, on the other hand, occurs when an issue has evolved into a well-characterized threat, with sufficient evidence to indicate a potential or increasing hazard to public, animal, or plant health.

The identification system, adapted from the EFSA methodology^17^, focuses on distributing information to stakeholders (government authorities, risk assessment bodies, researchers, consumers) to enable proactive responses to emerging risks.

### Data collection supported by text mining methods

Data collection is crucial and involves automated methods to handle the complexity and interdisciplinarity of emerging risks. These methods primarily target food safety news published online.

Information is also gathered through “soft” sources, such as expert communication via conferences and networks like the EFSA EREN Network, which provide valuable insights at the European level.

Information and data were collected from the Europe Media Monitor (EMM), which is a sophisticated news aggregation tool developed by the European Commission’s Joint Research Centre (JRC). It primarily focuses on monitoring and analysing media sources to provide timely and relevant information on diverse topics, including food safety. EMM aggregates data from a wide array of sources, including online news websites, blogs, and other digital media outlets, focusing on publicly available news content.

For the studied period, the „food safety” category was selected to ensure relevance. Textual data was retrieved weekly for which a workflow has been developed in KNIME software^18^ utilizing previously available RSS feed service of EMM. Similar approaches combining automated data collection and processing workflows (e.g. using KNIME or comparable tools) have been described in the context of emerging risk identification and early warning systems, including within the EFSA DEMETER project and in specific supply chains such as the dairy sector^2,19^. The purpose of the KNIME workflow is to automatically collect weekly news from the EMM and MediSys RSS sources. At the beginning of the process, the date range set in the metanode determines which period’s records will be processed. The workflow iterates through the predefined list of RSS feeds, reads the article metadata, and filters the items based on their publication date so that only those belonging to the selected week are retained. The KNIME workflow developed for this study is available at GitHub (https://github.com/Csorbasz/knime/blob/main/EMM%26MediSys_RSS_feed%20reader.knwf).

The resulting records are then standardized into a uniform structure, and the required fields are formatted. Exact duplicate entries are removed within the KNIME workflow, while highly similar or related articles are grouped during topic detection, which further reduces redundancy and supports efficient expert evaluation. Finally, the data is exported with the specified column structure into a CSV file in the designated target folder. The outcome is a standardized dataset containing the news for the given week. The workflow processes approximately 700–800 food safety news items per week, resulting in an estimated dataset of around 78,000 articles for the examined period. This served as weekly input for InfraNodus topic modelling software, which is a tool designed for text network analysis (web-based application, accessed in 2022–2023)^20^—the input table contains information on date, title and first 300 characters of each news. The collected data primarily consisted of English-language content. In cases where non-English articles were retrieved, automated translation to English was performed within the KNIME workflow to ensure consistency in downstream text analysis.

Steps and related algorithms embedded in InfraNodus software are the following:

- Data pre-processing:
  Text cleaning: Removal of unnecessary punctuation, stop words, and irrelevant content to ensure cleaner data for analysis.
  Tokenization: Splitting text into individual words or tokens, which can then be analyzed for frequency and co-occurrence.

b. Keyword Extraction:
  Term Frequency-Inverse Document Frequency (TF-IDF): Used to extract significant keywords from the text data, highlighting terms that are both frequent and uniquely significant within the dataset.

c. Network Analysis with InfraNodus:
  Building Text Networks: InfraNodus constructs a text network where nodes represent words, and edges represent co-occurrences of words within a certain context or window of text.
  Clustering and Community Detection: The software applies clustering algorithms to identify communities or clusters of related words, which correspond to different topics or emerging risks.
  Topic Identification: By examining the structure of the text network and the relationships between clusters, InfraNodus helps in identifying and visualizing emerging topics and trends within the food safety news data.

A feature of Infranodus is presenting the text network of the cases appearing during the week, enabling the viewer to identify critical nodes which may highlight emerging cases. Zooming into this network uncovers the cases contained which can be investigated more deeply. The visualization thereby assists the responsible screener in selecting the most relevant issues for further consideration in the screening system.

The analysis was performed using English language settings with stopword removal and lemmatization enabled. The network was constructed based on sentence-level adjacency, where nodes represent terms and edges represent their co-occurrence. Low-frequency terms were filtered to reduce noise. In addition, high-frequency but analytically non-informative terms (e.g. generic expressions such as “food” or “recall”) were excluded using frequency-based filtering (e.g. removal of top terms / nodes) and custom stopword settings within InfraNodus. This step reduced noise in the text network and prevented dominant but non-informative terms from disproportionately influencing the structure of the identified topic clusters. As a result, the analysis focused on more specific and informative signals relevant to emerging risk identification.

### Filtering process for the identification of emerging risks

The filtering process involves three stages:

- Pre-filtering: The Emerging Risk Identification (ERI) team screens collected data to select relevant emerging issues, discarding irrelevant ones.
- 1st Filtering: Potential emerging risks are identified using specific criteria. Issues not meeting these criteria are either dropped, revisited for more information, or communicated to stakeholders.
- 2nd Filtering: Risks needing further measures are evaluated using a scoring system based on factors like severity and management options. Issues below the threshold are monitored and periodically re-evaluated.

By analysing newly published information in a weekly manner by text mining methods, a vast amount of information is preprocessed before further screening by the experts. This saves much human time and effort, presenting focused and concise information for the subsequent steps.

However, the process cannot be entirely automated, as expert knowledge is essential for evaluating the reliability of information sources and making final decisions. Data analytical methods such as text mining of food safety news aid in screening, but human expertise validates and decides the fate of identified issues.

The ERI team, with diverse expertise in fields such as engineering, microbiology, and health economics, conducted pre-filtering and evaluated issues through 1st and 2nd filtering. Decisions were made by team consensus, with external experts consulted for specialized knowledge when necessary.

First, the text network constructed by InfraNodus was reviewed weekly by a designated member of the ERI team, with this responsibility rotating among team members. During this pre-filtering step, the assigned expert identified potential emerging issues based on initial judgement and domain knowledge. The aim at this stage was not to perform an in-depth investigation, but to capture all preliminary signals that could plausibly correspond to the concept of an emerging risk; therefore, uncertain or borderline cases were also retained.

The selected issues were then discussed by the full team, where members collectively assessed whether the cases should proceed to the first filtering stage. Following this initial team-level evaluation, the retained issues were assigned to domain experts for further investigation and structured evaluation according to the criteria described in our previous study.

Criteria at first filtering (validation as potential emerging risk) include the confirmation of the case as a new hazard (with or without significant exposure), or a known hazard with increased exposure or susceptibility. Criteria at second filtering (validation as emerging risk) include the scoring of the case with regard to its soundness, timeliness, spatial spread, severity, and the existence of suitable risk management measures in place.

Based on these evaluations, final validation of emerging risks or drivers, as well as decisions on further actions, were established through team consensus. During this process, domain experts played a key role in supporting or challenging the initial assessments, and disagreements were resolved through discussion. In cases where consensus could not be readily achieved, the final decision was made by the team supervisor, who is also a member of the EFSA EREN Network. Drivers of emerging risks—while methodologically managed similarly—were also classified and labelled at this final step. The primary direct action of the ERI system is the communication of identified issues to relevant audiences (e.g. authorities, consumers, academia). Further consideration and potential risk management measures are taken by competent authorities or relevant stakeholders. For example, national authorities may decide whether to incorporate identified and forwarded issues into monitoring plans, while at the European level, EREN members and EFSA experts may further evaluate and prioritize the reported potential emerging risks.

Repeated or follow-up news items referring to the same issue were treated as part of an evolving case and linked to the originally identified issue. These follow-ups were accumulated over time and contributed to the continuous evaluation of the case. Updates were handled on a case-by-case basis: major developments (e.g. significant changes in exposure or risk characterization) triggered immediate updates and communication, while less critical developments (e.g. additional detections of already identified hazards such as per- and polyfluoroalkyl substances—PFAS compounds—in new samples) were incorporated periodically based on expert judgement. In addition, both newly identified issues and relevant follow-ups were included in regular reporting to the EFSA EREN Network, typically prior to its biannual meetings.

## Results and discussion

### Summary of emerging risks and drivers identified in 2022–2023

During the years 2022–2023, a total of 611 cases were identified from the collected news at the pre-selection stage in Infranodus. After thorough discussions of each of them, 164 emerging risks/drivers were identified with the operation of the presented emerging risk identification system. Table 1 provides an overview of the groups into which these cases have been classified by the team. Some of these groups have been marked as drivers, denoting an external or internal force affecting the food system which can influence, directly or indirectly, and shape the food system in a positive or negative manner^21^. The categorization presented in Table 1 follows a pragmatic approach, combining topic-based (e.g. contaminants, pathogens) and mechanism- or driver-based groupings (e.g. antimicrobial resistance, climate-related effects). This reflects the nature of emerging risks, which may be defined either by the type of hazard or by underlying processes influencing their emergence and evolution.

**Table 1:** Distribution of emerging risks and drivers identified in 2022–2023 across major categories and subcategories. Shares represent the proportion of all identified cases (n = 164).

Category	Subcategory	Type	Cases (n)	Share (%)
Chemical risks (total)	–	Risk	**45**	**27.4**
	Pesticide-related issues	Risk	18	11.0
	Metals	Risk	6	3.7
	PFAS	Risk	3	1.8
	Micro/nanoparticles	Risk	4	2.4
	Additives	Risk	2	1.2
	Dioxins	Risk	2	1.2
	Phthalates	Risk	1	0.6
	Other chemical contaminants	Risk	9	5.5
Nutrition-related findings (total)	–	Risk	**20**	**12.2**
	Health effects	Risk	17	10.4
	New types of food	Risk	3	1.8
New harmful pests or agents (total)	–	Risk	**17**	**10.4**
Consumption-related trends (total)	–	Risk	**15**	**9.1**
	Consumer-driven trends	Risk	9	5.5
	Global trends	Risk	3	1.8
	Fraudulent activities	Risk	3	1.8
News on known microbes (total)	–	Risk	**9**	**5.5**
Harmful food/feed incidents (total)	–	Risk	**3**	**1.8**
Sustainability drivers (total)	–	Driver	**48**	**29.3**
	Alternatives to animal-based food	Driver	14	8.5
	Natural antimicrobials/preservatives/pesticides	Driver	11	6.7
	Sustainable packaging	Driver	7	4.3
	Sustainable substances/technologies	Driver	16	9.8
New technology drivers (total)	–	Driver	**7**	**4.3**
	GMO/NGT	Driver	1	0.6
	Nanotechnology	Driver	4	2.4
	Other new technologies	Driver	2	1.2

*Definitions: RISK: An emerging risk resulting from a newly identified hazard to which significant exposure may occur, or from an unexpected new or increased exposure and/or susceptibility to a known hazard^3^.

DRIVER: An external or internal force affecting the food system which can influence, directly or indirectly, and shape the food system in a positive or negative manner^21^.

GMO: genetically modified organisms; NGT: new genomic techniques.

*Chemical risks represented* a major focus, encompassing pesticides, metals, PFAS, micro/nanoparticles, additives, dioxins, phthalates, and other substances. Pesticide use and associated health/environmental risks gained significant attention. Glyphosate, widely authorized in regions like Europe, Canada, and the USA despite civil society concerns, was detected in human urine, raising health alarms^22^. Regulatory bodies faced intense scrutiny: the Pesticide Action Network (PAN) challenged the EU’s re-approval of cypermethrin against EFSA advice, while the US Environmental Protection Agency (EPA) faced lawsuits over reauthorizing dicamba despite known risks. Pesticide residues were pervasive, with EFSA reporting over a third of cereal products contaminated^23^, and French authorities identifying numerous residues in drinking water. Alarmingly, certain EU pesticides were found to contain PFAS (“forever chemicals”), and PAN Europe documented rising pesticide levels in French produce. Global increases in use (e.g., Costa Rica, Brazil:^24^) and emergency authorizations of hazardous pesticides (e.g., neonicotinoids in the UK, chlorpyrifos in Italy) further compounded concerns. Beyond pesticides, risks included lead contamination (chocolate, spices, breast milk), PFAS, microplastics, and other chemicals like TiO₂ (banned in the EU), nitrosamines^25^, sulfites, bisphenol-A (BPA)^26^, mineral oils, and smoke flavourings.

*New results from nutrition studies* explored links between diet and chronic diseases. Taurine was investigated for anti-aging potential^27^. Sugar intake was linked to kidney stones, while sweeteners faced controversy: erythritol was associated with cardiovascular risks and sucralose with immune effects^28^, and aspartame’s cancer link was debated. The World Health Organization (WHO) advised against non-sugar sweeteners for weight control. Cadmium exposure correlated with type 2 diabetes risk^29^, and excessive iron intake with parkinsonism. Early life nutrition was critical; Canadian research linked infant antibiotics to higher allergy risks^30^, and the US Food and Drug Administration (FDA) warned about probiotic risks for preterm infants.

*New harmful pests/agents* were identified. *Balantioides coli* was found in wild cervids in Portugal. Theileria parasites, spread by invasive Asian long-horned ticks posed threats. The Marburg virus caused outbreaks in Ghana, signaling its spread in West Africa^31^. A novel rustrela virus caused “staggering disease” in European cats^32^. Highly pathogenic avian influenza (H5N1) impacted minks in Spain^33^, and Alongshan virus (a Jingmenvirus) was detected in German ticks, with evidence of transmission to animals^34^.

*Consumption-related trends* highlighted emerging risks. Practices like poppy seed tea consumption, rising crocodile meat demand in Thailand, and “dumpster diving” due to economic pressures were noted. Home-roasting of cocoa beans for artisanal chocolate, driven by taste preferences, risked elevating acrylamide intake^35^.

*News on known microbes* focused on antimicrobial resistance^36–38^ and parasites in food chains^39,40^. **Harmful food/feed** incidents included tara flour illnesses in the US (FDA investigation concluded without definitive source) and risks from consuming sea cucumbers, which bioaccumulate heavy metals^41^.

While the previous report presented the main types of identified emerging risks, drivers are discussed separately due to their distinct conceptual role within the analytical framework. It is important to note that drivers identified in this study are not considered risks themselves, but rather underlying factors that influence the emergence, evolution, or amplification of risks. Distinguishing between risks and drivers, as presented in Table 1, allows for a clearer interpretation of causal relationships and supports a more structured assessment of emerging issues within the food chain.

*Main drivers* centered on **sustainability**, reflected in 48 identified cases across four areas: (1) **Alternatives to animal-based food** (veganism, fungi^42^, insects, plant-based meats, and cell-based meats, (2) **Natural antimicrobials/preservatives/pesticides** (seaweed, insect extracts^43^, essential oils^44^, salicylaldehyde^45^), (3) **Sustainable packaging** (silk coatings^46^, antioxidant/antibacterial materials^47^), and (4) **Sustainable substances/technologies** (using waste^48^, grass-derived starch, precision fermentation for honey/palm oil alternatives). **New technologies** like the EU’s proposed deregulation of New Genomic Techniques (NGTs) and nanomaterials for contamination control^49^ were also prominent, though their safety requires careful assessment.

The above description of the emerging food chain safety risks and drivers from 2022–2023 provides an illustration of the critical points where constant awareness is needed. Overall, the identified emerging risks can be grouped into several recurring patterns across domains. A substantial proportion of cases are associated with chemical hazards (e.g. pesticides, PFAS, microplastics), highlighting concerns related to persistent, cumulative, and low-dose exposures. Biological risks (e.g. pathogens, parasites, antimicrobial resistance) also represent a major group of cases, often linked to environmental conditions, globalization, and wildlife–livestock–human interfaces.

In addition, several cases are connected to innovation- and sustainability-driven transformations in the food system, including alternative proteins, novel ingredients, and new processing or packaging technologies. These developments introduce uncertainties related to exposure pathways, toxicological profiles, and long-term health impacts. These findings indicate that emerging risks are shaped by broader systemic drivers such as environmental change, technological innovation, and evolving consumption patterns, highlighting the need for integrated and forward-looking monitoring approaches.

Our system is a tool enabling us to supply all interested stakeholders with relevant and up-to-date information. We published news about these issues on our websites in 140 cases. The responsible Hungarian food safety authority was informed in 20 cases. Finally, information about 71 cases was presented to the regular meetings of the EFSA EREN Network.

### Comparison with previous years’ emerging topics

Table 2 presents a comparison between the identified groups in our present and preceding^16^ study period. The subjectivity of this comparison with regard to case numbers is noted, as each emerging risk/driver validation was based on expert judgement. Still, the change in numbers can provide interesting interpretations with regard to the evolvement of cases.

**Table 2:** Temporal evolution of emerging risk topics between 2020–2021 and 2022–2023. The table compares the number of cases assigned to comparable thematic categories across the two study periods. Relative change expresses the proportional increase or decrease in the number of identified cases.

Topic	2020–2021 cases	2022–2023 cases	Absolute change	Relative change (%)
Chemical contaminants/chemical risks	3	45	+ 42	+ 1400*
Sustainability-related issues/sustainability drivers	7	48	+ 41	+ 586
Technological innovation/new technology drivers	9	7	− 2	− 22
Emerging microbes/harmful pests or agents	6	17	+ 11	+ 183
Consumer trends	5	15	+ 10	+ 200
Nutrition-related findings	–	20	New category	–
Total identified cases	58	164	+ 106	+ 183

*Relative percentage changes are presented for illustrative purposes and may reflect increases from low baseline values.

A comparison of the two study periods reveals substantial changes in the distribution and intensity of emerging risk cases. As shown in Table 2, the total number of identified cases increased markedly from 58 in 2020–2021 to 164 in 2022–2023. The most pronounced growth occurred in chemical risks and sustainability-related drivers, which together account for the majority of detected cases in the recent period.

The sharp increase in chemical risk cases likely reflects growing regulatory scrutiny and scientific attention to contaminants such as pesticides, PFAS, and microplastics, although this increase is partly influenced by low baseline values in the earlier period. Several high-profile regulatory actions and risk assessments during the study period may also have amplified the visibility of these issues in the monitored information sources. At the same time, the strong growth of sustainability-related drivers highlights the ongoing transformation of food production systems, including the development of alternative proteins, novel packaging materials, and bio-based technologies.

In addition, nutrition-related findings appeared as a new topic category in the recent monitoring period. This development may reflect increasing public and scientific attention to the links between diet and chronic disease, as well as the growing role of nutrition-related narratives in food safety communication. Overall, these trends suggest that emerging risk monitoring increasingly captures cases and patterns associated both with regulatory challenges and with innovation-driven changes within the food system. Together, these trends reflect three major transformation processes affecting the food system: increasing regulatory attention to chemical contaminants, rapid sustainability-driven innovation, and growing societal focus on diet-related health risks.

### Distinction between food fraud detection and emerging risk identification

Although food fraud is not a primary focus of the present system, certain cases may be captured in the emerging risk identification system, irrespective of whether they originate from intentional or unintentional processes. It is important to note that targeted food fraud identification typically requires different methodological approaches, focusing on the detection of intentional deception and often relying on specialized analytical, intelligence, or traceability tools. In this context, previous studies have demonstrated that media monitoring systems based on the EMM infrastructure can be specifically adapted to identify food fraud cases through targeted keyword strategies and filtering approaches^50,51^. In contrast, the present system does not apply such targeted adaptations, as its objective is to capture a broad range of emerging risks across the food system rather than focusing on specific predefined topics.

Within this framework, fraud-related early signals are treated uniformly during identification process and communication, as the system does not differentiate based on intent at this stage. At the level of risk management, however, different competent authorities or regulatory pathways may be involved depending on the nature of the issue.

### Strengths, limitations and future directions

The presented framework illustrates the potential of hybrid monitoring systems that combine automated text mining of large information streams with expert evaluation to support the early identification of emerging risks and their drivers in complex food systems.

The monitoring system processed weekly food safety news collected from the Europe Media Monitor, resulting in a large textual dataset covering the 2022–2023 period. The amount of news gathered reached about 700–800 individual pieces weekly, all of which were processed within the workflow.

However, this work also encountered some challenges. While traditional text mining methods (e.g., InfraNodus-based TF-IDF and network analysis) proved effective in preprocessing large datasets and mapping broad topic clusters, they faced limitations in semantic granularity. These tools excelled at filtering “noise” (high amount of unstructured data) from sources like the EMM and distilling weekly news into actionable inputs for expert review, yet struggled to capture contextual nuances. This gap highlighted the need for more sophisticated analytical frameworks capable of interpreting subtle linguistic patterns and evolving risk trajectories.

To address these challenges, we explored the BERTopic framework, which dynamically tracks temporal shifts in discourse and interprets semantic relationships without manual intervention. Preliminary integration into our workflow in 2024 demonstrated promising gains in topic coherence and scalability, though computational demands and integration complexity remain hurdles.

While structured procedures and team-based consensus help to mitigate the influence of subjectivity, the system still relies on expert judgement in the selection and evaluation of potential emerging risks. This may introduce some variability in interpretation; however, the overall approach supports consistent and transparent decision-making.

Looking ahead, further development of the presented framework may benefit from the integration of recent advances in natural language processing. In particular, embedding-based language models and transformer architectures offer promising opportunities to capture semantic relationships and contextual signals in large textual datasets more effectively than traditional keyword-based approaches. Integrating such methods into emerging risk monitoring workflows could enable more sensitive detection of weak or early signals, facilitate the tracking of evolving risk narratives over time, and further strengthen data-driven food safety intelligence. Exploring the practical integration of these approaches into expert-supported monitoring systems therefore represents an important direction for future research.

### Validation of emerging risk identification systems

The evaluation of emerging risk identification systems presents specific challenges, as the objective is not to predict predefined outcomes but to detect signals and evolving patterns in a forward-looking context. Consequently, ground truth is often not directly observable at the time of detection. In line with approaches used in early warning and event-based surveillance systems (e.g. WHO^52^; ECDC^53^), validation therefore relies on a combination of expert assessment, temporal consistency of detected cases, and practical usefulness within real-world risk governance frameworks.

In such systems, expert judgement plays a central role in interpreting and validating signals and potential emerging risks, while continuous monitoring enables their assessment persistence and evolution over time. At the same time, retrospective approaches (e.g. backcasting) may provide complementary insights, although their interpretation remains limited due to intervention effects and partially observable outcomes.

Further development of evaluation approaches may be supported by increasing the role of data-driven and automated components within such hybrid systems, enabling more systematic tracking, comparison, and quantification of detected signals.

Although systems such as the Rapid Alert System for Food and Feed (RASFF) provide valuable information on food safety events, it should be noted that they primarily capture known and confirmed risks, representing the short-term, early warning component of the emerging risk horizon (FAO^54^; Farkas & Jóźwiak^55^). In contrast, emerging risk identification focuses on the detection of early signals of risks that may materialize in the medium term.

While direct comparison between these systems is methodologically challenging due to their differing objectives and time horizons, RASFF-based analyses may provide complementary insights, particularly in the evaluation of longer-term trends or changes in exposure patterns. Such approaches could therefore support future methodological developments in emerging risk identification.

## Conclusions

The identification of emerging risks in complex food systems is challenged by the increasing volume and heterogeneity of available information. The objective of this study was to assist emerging risk identification experts by filtering and visualizing various clusters of potential emerging issues. It is important to note that topic detection methods will not predict the specific emerging issues or forecast the future. However, they are suitable for the quick analysis of large text corpora, effectively reducing noise and distinguishing “important” information from “less important” information.

The systematic identification of emerging risks in the food chain during 2022–2023, facilitated by our phased process management system and text mining methodologies, as a hybrid monitoring system, underscores the dynamic nature of global food safety threats. A total of 164 emerging risks and drivers were identified, reflecting heightened vulnerabilities driven by regulatory shifts, environmental pressures, and evolving consumer behaviors. Chemical risks dominated this landscape, and concurrently, sustainability drivers surged.

Ultimately, the synergy of structured process management and text mining offers a structured framework for anticipatory food safety governance. By supporting more proactive and structured monitoring, this framework not only aids to mitigate the possible effects of emerging risks, but also contributes to fortifying global food systems against an era of unprecedented disruption. In an increasingly complex global food system, monitoring approaches that integrate automated information processing with expert knowledge may play an important role in supporting anticipatory food safety governance.

## Data Availability

The datasets used and/or analyzed during the current study are available from the corresponding author on reasonable request.

## Code availability

The KNIME workflow developed for the analyses in this study is publicly available at GitHub: https://github.com/Csorbasz/knime/blob/main/EMM%26MediSys_RSS_feed%20reader.knwf
